# Associations of genetic polymorphisms with boldness, stress response, and route efficiency in homing pigeons (*Columba livia*)

**DOI:** 10.1016/j.physbeh.2025.115211

**Published:** 2026-03-01

**Authors:** Sherif I. Ramadan, Tin Hang Hung, Mathilde Delacoux, Fumihiro Kano, Miho Inoue-Murayama, Dora Biro

**Affiliations:** a Faculty of Veterinary Medicine, Benha University, Moshtohor 13736, Egypt; b Faculty of Veterinary Medicine, Delta University for Science and Technology, Mansoura 11152, Egypt; c Department of Biology, University of Oxford, South Parks Road, Oxford OX1 3EL, UK; d Centre for the Advanced Study of Collective Behaviour, University of Konstanz, 78464 Konstanz, Germany; e International Max Planck Research School for Quantitative Behavior, Ecology and Evolution, 78315 Radolfzell, Germany; f Wildlife Research Center, Kyoto University, Kyoto 606-8203, Japan; g Department of Brain and Cognitive Sciences, University of Rochester, Rochester, NY 14627-0268, USA

**Keywords:** Pigeon, Genetic polymorphism, Boldness, Navigation, Stress response

## Abstract

Animals vary consistently in traits such as boldness and stress reactivity, which influence fitness, movement, and social dynamics. Identifying genetic variants linked to these behaviours can clarify proximate mechanisms and evolutionary trade-offs. Dopaminergic (DRD4) and serotonergic (TPH2) pathways are known to modulate exploration and emotionality, while LDHA contributes to neuroenergetics and endurance, yet their combined role in shaping behavioural diversity remains unclear. Here we show, in 137 homing pigeons, that a SNP at position C382T in *DRD4* and T185A in *TPH2* are significantly associated with boldness. Birds with *DRD4* T/T and *TPH2* T/A genotypes emerged more quickly from the shelter. We also found that *DRD4* C/C and *TPH2* T/A genotypes were associated with slower recovery from acute social isolation stress, quantified by eye infrared thermography. In addition, a microsatellite polymorphism in *LDHA* intron 6 interacted with the presence of a mirror companion, suggesting a genotype-dependent effect of social buffering. These findings provide evidence that common genetic variants in neuromodulatory and metabolic genes contribute jointly to behavioural and physiological syndromes in birds, and social context can interact with genotype to influence stress resilience. We demonstrate how candidate markers are useful in exploring the ecological and evolutionary consequences of personality variation.

## Highlights

• DRD4 C382T and TPH2 T185A variants predict boldness in homing pigeons, where DRD4 TT and TPH2 TA genotypes exit shelters faster.
• DRD4 CC and TPH2 TA genotypes recover more slowly from isolation stress.
• Different genotypes in LDHA intron-6 microsatellite interacts differently with mirror cues to modulate stress.
• Genotype and social context jointly shape behavioural syndromes.

## 1 Introduction

Since the domestication of wild rock doves (*Columba livia*) at least 5000 years ago in the Middle East, breeders have been using artificial selection to develop strains of pigeons with improved homing performance and preferred appearances [1]. This long history of artificial selection makes pigeons a valuable model for studying the relationship between behaviour and adaptation. Behavioural variation – differences in how individuals respond to environmental challenges and opportunities – has long been recognised as a key factor influencing fitness [2]. Such variation can affect animals’ ability to exploit novel resources or cope with changing conditions, with important ecological and evolutionary consequences [[3], [4], [5]]. Recent work suggests that behavioural phenotypes may themselves be under selection, providing adaptive advantages as a function of context and shaping evolutionary trajectories [6]. When the behavioural phenotype is consistent across time and ecological context, it can be used to define an animal’s personality [7].

One of the most studied aspects of personality in animals is boldness [8]. Boldness is mainly expressed in the form of exploratory behaviours and relates to the ability to overcome neophobia when emerging from a safe shelter or exploring novel environments [9]. The bold-shy continuum presents an evolutionary trade-off. Among birds, for example, bold individuals may have higher reproductive success and better homing ability with more efficient access to novel resources [10]. They can also be more influential in collective decision-making: in the case of homing pigeons, for example, bold individuals are responsible for initiating a greater proportion of a flock’s directional changes than shy individuals [[11], [12], [13]]. However, shy birds have been shown to be better at evading predators and injury during exploration [10]. This personality trait has also been found to correlate with cognition, with bolder individuals shown to be better at learning new tasks, while performing less well at reversal learning [[14], [15], [16], [17], [18], [19]], although this may depend on the context [[20], [21], [22]]. Boldness appears to be a repeatable, consistent trait in birds [23], and has been found to be heritable and hence subject to change in response to artificial selection [24,25]. The trade-off is likely the ultimate factor facilitating the persistence of the bold-shy continuum in natural populations [26].

Boldness is often linked to other dimensions of personality and phenotypic traits. In particular, response to stress is found to be prominent and variable in social animals when tested in the context of social detachment or isolation [27]. Bold birds exhibit relatively lower stress responses, characterised by lower levels of the stress hormone corticosterone [28]. In contrast, shyness in birds is associated with more acute hypothalamic-pituitary-adrenal (HPA) axis activity [29]. While long-term stress was traditionally measured using faecal cortisol, advances in infrared thermography have made the non-invasive study of acute stress possible, facilitating its investigation at a much finer scale [30]. In addition, leveraging data from the prevalent sport of pigeon racing [[31], [32], [33], [34]] has shown correlations between boldness and navigational performance. Neophobic, shy pigeons performed less spontaneous exploration of their surroundings, flew slower, and took longer homing routes compared to bold birds [12,13].

Given the heritability and potential linkage of these traits, a better understanding of the underlying molecular genetic basis can yield important insights into the evolution of behaviour and personality and ultimately adaptation [35]. Individual variation can have several dimensions and the behavioural response in a given situation is partly dependent on genetic factors [36]. Genetic polymorphisms in candidate genes that govern various behavioural traits have been studied in different animals [3,35,37].

Dopamine receptor D4 gene (*DRD4*) is part of the dopaminergic and the serotonergic neurotransmitter system, which modulates the amygdala-connected circuitries that are crucial in emotional modulation and response to stress and anxiety-related behaviours [38]. Dopamine receptors are implicated in many neurological processes, including motivation, pleasure, cognition, memory, learning, and fine motor control, as well as modulation of neuroendocrine signalling [39]. Polymorphism of *DRD4* has been linked to novelty seeking, exploratory behaviour, and navigation ability [[40], [41], [42], [43]]. Given that bold individuals show more intense exploratory, novelty-seeking, and locomotor behaviour [44], we hypothesize a link between polymorphism of *DRD4* and boldness expressed at the behavioural level. Furthermore, studies have also shown that polymorphism of *DRD4* can affect individual responses to stress [45].

Tryptophan hydroxylase 2 gene (*TPH2*) affects the synthesis of serotonin neurotransmitters through the tryptophan hydroxylase. Serotonin receptors occupy strategic cellular and sub-cellular locations in the hippocampus and have long been known to be involved in behaviours related to boldness [46,47]. The effects of TPH2 in non-model animals have been studied in wild primates – for example, in the context of vigilance behaviour in monkeys [48] – but never in birds. TPH2 affects stress response, as well as locomotory and exploratory behaviour [49].

Lactate dehydrogenase A (LDHA) catalyses the interconversion of pyruvate and lactate, with nicotinamide adenine dinucleotide as a coenzyme [31,50]. Lactate’s function is now being considered as a neuroenergetic and signaling molecule [51]. Lactate is also known as an important cellular fuel, including in hippocampal nerve cells, through its role in the astrocyte-neuron lactate shuttle. The hippocampus has a well-known role in cognition, particularly in long-term memory formation and spatial navigation [52]. Moreover, lactate plays a role in the stress response through its antidepressant effect which is achieved by the activation of protein kinase C and upregulation of tryptophan hydroxylase expression, which eventually leads to enhanced serotonin and dopamine levels in the brain [53]. LDHA activity is also found to be associated with homing ability and has been subjected to artificial selection in top racing pigeons [54].

The overarching goal of the current study is to test for possible associations between three candidate genes, *DRD4, TPH2*, and *LDHA*, and three dimensions of individual behavioural variation, namely boldness, stress response, and navigational performance. We hypothesise that polymorphisms in *DRD4* and *TPH2* would be related to differences in boldness and stress response, while polymorphisms in *LDHA* would be associated with differences in navigational performance. In the stress response assays, we further explore if stress response could be buffered by a mirror companion and if such mitigation is differential across genotypes.

## 2 Materials and methods

### 2.1 Subjects

We tested 137 homing pigeons (*C. livia*), bred and housed in two adjacent pigeon lofts at The John Krebs Field Station, University of Oxford, Wytham, UK. The ages of the birds ranged between one and 20 years. The sex of the birds was identified by genotyping chromo-helicase DNA-binding gene (*CHD*) as detailed in the Genotyping section. There were 75 males, 60 females, and 2 undetermined due to low-quality DNA sampling. Although normally free-ranging, due to the implementation of an Avian Influenza Prevention Zone just prior to our study, the birds had no access to the outside during the study. Food and water were provided ad libitum during daily provisioning, and birds were routinely weighed and inspected for health. Metadata for all birds are available in the Supplemental Data.

### 2.2 Genotyping

Feather samples were collected from all 137 individuals. DNA was extracted from feather roots using the QIAGEN DNeasy Tissue Kit (QIAGEN, Valencia, CA, USA).

We chose the widely polymorphic regions known to affect biochemical synthesis which are the most informative for association testing in our study. For genotyping of *DRD4* exon 3 and *TPH2* (part of exon 10 + intron 10 + exon 11 + part of 3 UTR), primer pairs were designed using Primer 3 plus Version 2.3.6 software [55], based on the registered pigeon *DRD4* (NC_088606.1and) and *TPH2* (NC_088602.1) as shown in Table 1. PCR was performed in a 15 μl reaction mix containing 20 ng of genomic DNA, 2x PCR buffer, dNTPs at 400 μM, each primer at 0.3 μM, and 0.5 U of LA-*Taq* DNA polymerase (TaKaRa, Shiga, Japan). The PCR cycle conditions are shown in Table 1. The amplified products were purified using a PCR purification kit (Roche, Mannheim, Germany), and the resultant products were sequenced using the same primers with Big Dye Terminator ver. 3.1 Cycle sequencing kit (Applied Biosystems, Foster City, CA, USA) according to the standard protocol, and electrophoresed on an ABI PRISM 3130xl sequencer (Applied Biosystems, Foster City, CA, USA). BLAST software [56] was used for sequence identification and confirmation. FinchTV 1.4.0 (Geospiza, Inc., Seattle, WA, USA; http://www.geospiza.com), MEGA 11 [57], and Bioedit 7.0.5.3 [58] software were used for sequence alignment and polymorphism detection.

**Table 1:** Primer sequences, type of mutations, and PCR conditions for genotyping of *DRD4, TPH2, LDHA*, and *CHD* genes in homing pigeons.

Gene	length	Type of Mutation	Primer sequence	PCR condition
*DRD4*	415 bp	SNP C382T (exon III)	F 5′-CCTACGGCAGCTGATCCTTA-3′ R 5′-CTTGAGGTGGGGATAGGAGA-3′ (This study)	95 °C for 30 s, 59 °C for 15 s, 74 °C for 15 s
*TPH2*	720 bp	SNPs T185A (intron X) C277T (exon XI)	F 5′-CTTTCTGACAAGGCCAAGGT-3′ R 5′-CCTAATGAAATCAGCAACACCA-3′ (This study)	95 °C for 30 s, 59 °C for 30 s, 74 °C for 30 s
*LDHA*	645 bp	microsatellite (TTTAT)_3__–__5_ (intron VI)	F 5′-CCTGAAGGCTCTTCATCCAG-3′ R 5′-TTGGGTGCACTCTTCTCAAA-3′ (Ramadan et al. 2018)	95 °C for 30 s, 55 °C for 45 s, 74 °C for 30 s
*CHD*		microsatellite	F 5′-CTCCCAAGGATGAGRAAYTG-3′ R 5′-ATGGAGTCACTATCAGATCCAG-3′ (Lee et al. 2007)	95 °C for 30 s, 57 °C for 45 s, 74 °C for 60 s

For genotyping of *LDHA* (part of exon 5 + intron 5 + part of exon 6) (Supplementary Fig. 1) and *CHD* (Supplementary Fig. 2), their microsatellites were amplified using fluorescently labeled (6-FAM) forward primers (Table 1) [34]. PCR reaction mixture was 10 μL, including 20 ng of genomic DNA, 2x GC buffer I, dNTPs at 400 μM each, forward/reverse primers at 0.3 μM, and 0.5 U of LA-*Taq* DNA polymerase (TaKaRa, Shiga, Japan). Thermocycling conditions are shown in Table 1. PCR products were electrophoresed on an ABI 3130xl DNA Sequencer (Applied Biosystems, Foster City, CA, USA). Fragment sizes were estimated based on the fluorescently labeled forward primer in GENE MAPPER (Applied Biosystems, Foster City, CA, USA)**.** Estimations of genotypic and allelic frequencies, heterozygosity (*H_E_*), number of effective alleles (*N_E_*), and Hardy-Weinberg equilibrium were carried out using GENALEX version 6.0 [59].

### 2.3 Behavioural tests

#### 2.3.1 Boldness

The widely used “emergence test” of boldness was used to determine each bird’s behaviour on the bold-shy axis: birds were individually placed in a shelter, and we measured the latency before they voluntarily exited this shelter and entered a novel, open space. The shelter was made of a cardboard box (34 × 27 × 27 cm), and the open space was a 27-cm wide and 140-cm long passage with wood-board sides [13]. A small pile of multigrain-mixed food was situated at the far end of the passage. The birds’ daily feeding routine was shifted during the experiment, such that they were only fed in lofts after the experiments were finished every day. At the start of each test, a bird was placed in the shelter, covered in front by an opaque shutter. The bird was then allowed to habituate for 3 min, followed by the removal of the shutter. The bird was then left undisturbed, and the experimenter remained silent and out of the bird’s view. The time taken by the subject to emerge from the shelter was recorded, and once emerged, the bird was immediately removed from the apparatus and returned to the loft. If a bird did not emerge within 10 min, the trial was concluded and the bird was returned to the loft. No bird was allowed access to the food at the end of the passage, to avoid reinforcing the emergence. Each test was filmed from above by a tripod-mounted video camera (HC-V520, Panasonic, Japan). Each bird was tested twice with an interval of 36 days between the two tests to quantify repeatability in the time taken for a given subject to emerge from the box. All 137 pigeons participated in our boldness assays.

#### 2.3.2 Stress response to social isolation

Birds were caught from the loft and brought indoors inside a transit box, followed by a minimum of 20-minute habituation time outside the test room, where they maintained social contact with their conspecifics inside the transit box. After the habituation time had elapsed, birds were brought individually into the test room with constant ambient lighting. The bird was immediately introduced into a novel cage, which comprised two equal-sized compartments separated by a wire-mesh divider. The focal bird was placed in the near compartment with no perch, food, or water provided and left undisturbed for 10 min (i.e., they experienced social isolation in a novel environment). The interior space was deliberately constrained to the extent that the bird could stand and turn only a limited amount. In this no-mirror condition, the far compartment was empty. For the duration of the 10-minute test period the bird was videotaped by a thermal imaging camera (FLIR T620bx, FLIR Systems, United States) from a set distance of 1 m. The experimenter was hidden behind a plain white cloth and controlled the photography equipment remotely using the FLIR Tools software to minimise any effect of observer presence.

Video footage was subsequently analysed with a focus on changes in maximum eye temperature, a reliable indicator of stress in birds when using infrared thermography [30], as temperature varied in different parts of the eye. Maximum eye temperatures of the birds were obtained from the thermal videos using the software FLIR Tools, at one-minute intervals. Since birds were not fully restrained and hence their eyes were not always within the camera's view, a time window of 10 s either side of the one-minute mark was allowed for camera adjustment to capture the eye image.

Following completion of the above, the test was repeated for each subject, with the only modification being that a mirror (297 mm × 210 mm) was placed at a distance of 20 cm in front of each pigeon, in the far compartment. We used a flat mirror as a standardised visual social cue to approximate social presence without physical contact, which [60]has been shown to attenuate isolation-related stress in other bird species such as European starlings [61] and domesticated chickens [62]. As such, we treat the mirror condition as a pragmatic social-cue manipulation. 134 of our total of 137 birds participated in the two social isolation tests.

#### 2.3.3 Route efficiency

We quantified the route efficiency of a subset of our subjects using data from a previous study [13]. GPS-mounted birds were released individually from a novel site (Church Hanborough: 51°48′44.3″N, 1°22′38.3″W; distance to home: 5.2 km, direction to home: 128°) and their navigational performance was quantified from GPS data in terms of the ratio of straight-line distance to actual flight distance (termed *route efficiency*). Higher route efficiency implied better navigational performance by a given bird. 20 of our total of 137 birds participated in these navigation trials.

### 2.4 Data analyses

All statistical analyses were performed in R Studio, using established and publicly available statistical packages, as detailed below.

#### 2.4.1 Boldness

The distribution of times taken by subjects to emerge from the shelter (latency) were non-Gaussian and also right-censored given the 600-second limit on each trial. To test for repeatability in the measure of boldness, we thus fitted two different models using the rpt() function (rptR package). The first model was binary and predicted whether or not the individual emerged from the box, and the second tested the latencies of individuals that emerged both times using a Gaussian distribution. For both models, trial number, age, weight and sex of the subject were added as control variables. To test for the relationship between the different genes and the boldness response, we fitted a Cox proportional hazards regression model on the latency and emergence status with the fixed effect of genotypes and the random term of individual ID. To rule out the confounding effects of age, weight, sex, or trial number, we added each term to the regression model and used a likelihood ratio test to compare the models.

#### 2.4.2 Stress response to social isolation

Maximum eye temperature was measured in all birds for 10 min following transfer to the experimental cage. The mean change in maximum eye temperature over the test period was computed for each genotype to determine the effect of genetic polymorphism on stress-induced eye temperature change. For some subjects, there were a few time windows when no eye image could be captured (1.21 % of the dataset), usually because the bird turned its head away for a short period, and these incomplete time series were completed by R package imputeTS by interpolation.

We first tested if different genotypes had different baseline temperatures at the beginning of the social isolation test. To do so, we ran a linear mixed model predicting the temperature at timestep zero by the genotype of the individual (testing the different genes in three different models). We added the age, weight, sex as well as a random effect of the individual ID as control variables.

Second, we tested if the evolution of the eye temperature over time in response to the social isolation was different between genotypes. We analysed the evolution of the temperature by subtracting the baseline temperature at timestep zero from each timestep’s temperature. We then checked the presence of a difference in the evolution of the temperature by testing the effect of the interaction between timestep and genotype in a set of linear mixed models (lmer() function from lme4 package). In all models, the evolution of the temperature over the timesteps was approximated using a sigmoid curve (SSlogis() function from the stats package). The first model included a triple interaction timestep*genotype*condition (presence or absence of a mirror) to also account for a potential differential effect of the genotype on the evolution of the temperature depending on the presence of the mirror. As this interaction was not found to be significant, we used a second model testing the interactions (timestep*genotype, timestep*mirror, and mirror*genotype) separately. We tested all three genes in different models, and added the age, weight, sex as well as a random effect of the individual ID as control variables in all models.

We verified all model assumptions by checking the distribution of the residuals in diagnostic plots (histogram of the residuals, qq-plot and plot of the residuals against fitted values).

#### 2.4.3 Route efficiency

The means of individual route efficiencies were computed for each genotype and fitted to a Generalized Linear Model for one-way ANOVA to determine the effect of genetic polymorphism on individual flight characteristics during solo homing.

## 3 Results

### 3.1 Genotyping

We identified one SNP C382T in the *DRD4* exon 3, two SNPs T185A in intron X and C277T in exon 11 of the *TPH2*, and one microsatellite in *LDHA* intron 6 gene resulted in three alleles (*S* = 635 bp, *M* = 640 bp, *L* = 645 bp) and six genotypes (SS, SM, SL, MM, ML, and LL). The two SNPs in *TPH2* were in complete linkage disequilibrium. Therefore, we only took T185A for subsequent analyses of the *TPH2*. Both mutations in *DRD4* and *TPH2* were found to be synonymous and did not alter the amino acid sequence.

The genotypes and allele frequencies, expected heterozygosity (*H_E_*), number of effective alleles (*N_E_*), and Hardy-Weinberg equilibrium are presented in Table 2. Among the three investigated genes, the *TT* genotype of *DRD4, AT* of *TPH2*, and *MM* of *LDHA* showed the highest frequencies at 50.8 %, 46.2 %, and 26.2 % respectively. The highest allele frequency values were recorded for *T* allele of *DRD4* (70.4 %), *T* allele of *TPH2* (56.9 %), and *M* allele of *LDHA* (48.5 %). *LDHA* showed the *H_E_* (0.632), and *N_E_* (2.698). A chi square test showed that our tested pigeon population was in Hardy-Weinberg equilibrium. The male-to-female sex ratio based on *CHD* genotyping was 1:1.24.

**Table 2:** Genetic polymorphism and summary statistics of *DRD4, TPH2*, and *LDHA*.

Gene	Observed genotype frequencies			Expected genotype frequencies			Allele frequencies		*H_E_*	*N_E_*	Hardy-Weinberg equilibrium	
											*X^2^*-test	*P* value
***DRD4***	**CC**	**CT**	**TT**	**CC**	**CT**	**TT**	**C**	**T**	0.419	1.715	0.452	0.501ns
	0.100	0.392	0.508	0.114	0.542	0.644	0.296	0.704				
***TPH2***	**AA**	**AT**	**TT**	**AA**	**AT**	**TT**	**A**	**T**	0.492	1.962	0.451	0.502ns
	0.200	0.462	0.338	0.241	0.638	0.421	0.431	0.569				
***LDHA***	**SS**	**SM**	**MM**	**SS**	**SM**	**MM**	**S**	**M**	0.632	2.698	3.988	0.263ns
	0.123	0.231	0.262	0.114	0.373	0.305	0.296	0.485				
	**SL**	**ML**	**LL**	**SL**	**ML**	**LL**	**L**	**-**				
	0.115	0.215	0.054	0.169	0.276	0.062	0.219	-				

H_E_ = Expected heterozygosity, N_E_ = effective number of alleles, ns = Not significant.

### 3.2 Boldness

We found significant repeatability in the pigeons’ likelihood of emerging from the box within 10 min between the two trials (*R* = 0.235, *p* = 0.004, *n* = 137). The latency to emerge from the box, however, was not significantly repeatable (*p* = 0.146, *n* = 74), despite a similar repeatability coefficient (*R* = 0.199). This might have been caused by the reduced number of observations, as only pigeons that left the box in both trials were considered in this test.

**Fig. 1:**
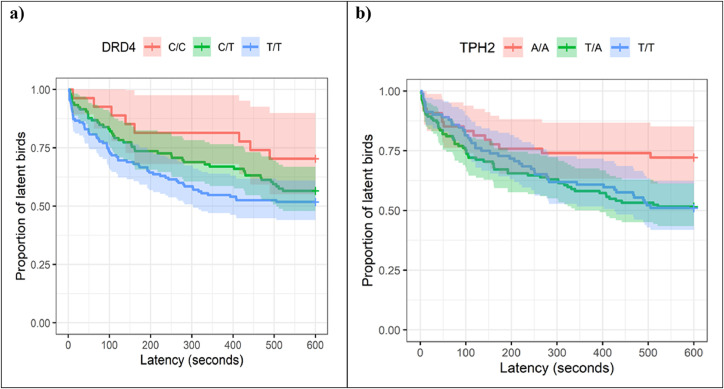
Effects of genetic polymorphism at **(a)***DRD4* C382T and **(b)***TPH2* T185A on boldness (latency to exit a shelter) in homing pigeons tested with Cox proportional hazards regression models (*n* = 137). The shaded areas denote 95 % confidence intervals.

In testing the effect of genotype of *DRD4*, likelihood ratio tests determined that none of the potentially confounding effects of age (λ = 3.20, df = 1, *p* = 0.073), weight (λ = 0.90, df = 1, *p* = 0.34), sex (λ = 0.33, df = 1, *p* = 0.56), and trial number (λ = 0.32, df = 1, *p* = 0.57) significantly improved the Cox proportional hazards regression model. The simple model showed a significant association between genotype of *DRD4* and latency to emerge from the box: when compared to the C/C genotype, C/T showed a small but insignificant decrease in latency (*z* = 1.58, *p* = 0.11) and T/T showed a significant decrease in latency (*z =* 2.34, *p* = 0.019). The mean latency for C/C was 490 s, C/T 425 s, and T/T 373 s. This implies that the point mutation from C to T allele in *DRD4* contributes to behavioural variation in homing pigeons, with the T allele associated with increased boldness as shown in Fig. 1a.

Similarly, in testing the effect of genotype of *TPH2*, likelihood ratio tests determined that none of the confounding effects of age (λ = 2.65, df = 1, *p =* 0.10), weight (λ = 3.09, df = 1, *p* = 0.08), sex (λ = 0.97, df = 1, *p* = 0.33), and trial number (λ = 0.24, df = 1, *p* = 0.62) significantly improve the Cox model. The simple model showed a significant association between genotype of *TPH2* and latency: when compared to the A/A genotype, T/A showed a small and significant decrease in latency (*z* = 1.98, *p* = 0.048) and T/T showed a small but insignificant decrease in latency (*z =* 1.82, *p* = 0.070) as shown in Fig. 1b The mean latency for A/A was 464 s, T/A was 384 s, and T/T 401 s, which implied that the heterozygous genotypes were most associated with increased boldness in homing pigeons. None of the main effects of genotype of *LDHA*, nor any of the control variables showed significant association with latency to emerge from the box (*p* > 0.05).

### 3.3 Eye temperature response to social isolation and mirror

**Fig. 2:**
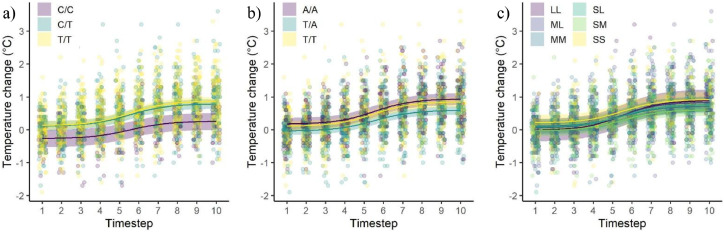
Eye temperature changes compared to the baseline (timestep 0) for each timestep for different genotypes of a) *DRD4*, b) *TPH2* and c) *LDHA* (*n =* 134). Regression lines were determined with other variables held constant, set to their mean values, and the ribbon shows the 95 % confidence interval (based on 10 000 simulations of the parameters). Individual datapoints are represented with horizontal jitter for visualization purposes.

**Fig. 3:**
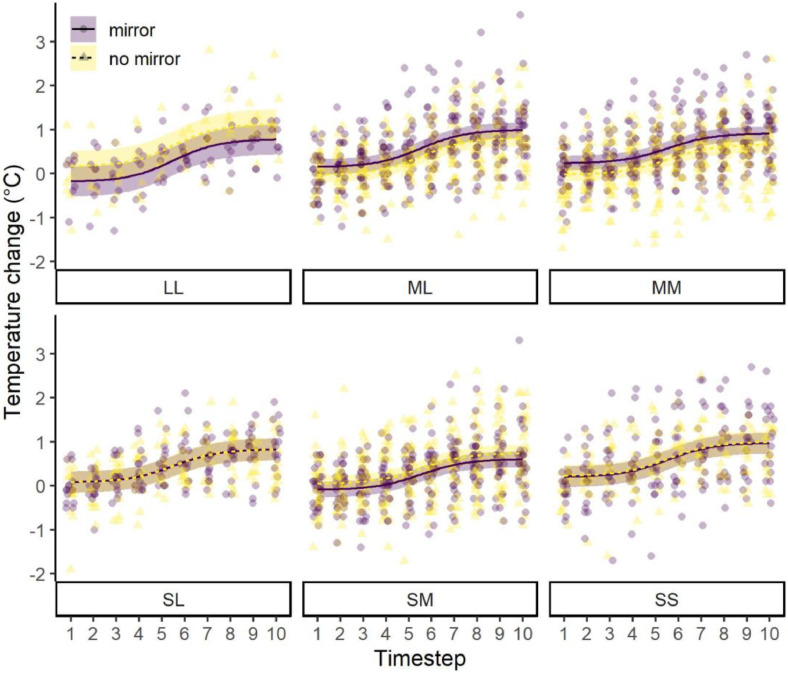
Eye temperature changes compared to the baseline (timestep 0) for each timestep for different genotypes of *LDHA* (*n* = 134). The data and regression lines from the trials including a mirror are shown in purple dots and plan lines, while those not including a mirror are shown in yellow triangles and dashed lines. Regression lines were determined with other variables held constant, set to their mean values, and the ribbon shows the 95 % confidence interval (based on 10 000 simulations of the parameters). Individual data points are represented with horizontal jitter for visualization purposes.

We first checked whether there was a difference in baseline temperature for the different genotypes using linear mixed models. We did not find any differences in temperature for *DRD4* (χ² = 0.433, df = 2, *p* = 0.805), *TPH2* (χ² = 1.553, df = 2, *p* = 0.460), and *LDHA* (χ² = 5.148, df = 5, *p* = 0.398). We then tested for a differential evolution of the temperature over the course of the trial depending on the presence of a mirror companion with a linear model including a triple interaction timestep*genotype*mirror. This triple interaction was not found to be significant for *DRD4* (χ² = 0.868, df = 2, *p* = 0.648), *TPH2* (χ² = 1.683, df = 2, *p* = 0.431), and *LDHA* (χ² = 5.105, df = 5, *p* = 0.403). We then tested the interaction between the genotype, the timestep, and the condition separately (timestep*genotype + mirror*genotype + timestep*mirror). The model including *DRD4* showed a significant effect of the timestep*genotype interaction (χ² = 6.730, df = 2, *p* = 0.035), with individuals with the C/C genotype showing a slower increase in temperature compared to the other two genotypes (C/T and T/T) as shown in Fig. 2a. The mirror*genotype interaction was not significant (χ² = 0.364, df = 2, *p* = 0.834). Similarly, the model including *TPH2* showed a significant effect of the timestep*genotype interaction (χ² = 6.079, df = 2, *p* = 0.048) as shown in Fig. 2b but no effect of the mirror*genotype interaction (χ² = 2.152, df = 2, *p* = 0.341). Lastly, the model for *LDHA*, although the trend for the timestep*genotype interaction was not statistically significant (χ² = 10.603, df = 5, *p* = 0.060; Fig. 2c), we found a significant effect for the mirror*genotype interaction (χ² = 79.568, df = 5, *p* < 0.001; Fig. 3). In addition, the interaction timestep*mirror was significant for all *DRD4* (χ² = 6.936, df = 1, *p* = 0.008), *TPH2* (χ² = 6.872, df = 1, *p* = 0.008), and *LDHA* (χ² = 6.424, df = 1, *p* = 0.011), with the mirror condition showing a steeper increase in eye temperature compared to the no-mirror condition.

### 3.4 Navigational performance

Due to the limited sample size of 20 birds, normality could not be assumed and, after logarithmic transformation, the distributions of route efficiency (*p* = 0.78) were still not normal (Shapiro-Wilk normality test). The Generalized Linear Model revealed no significant effects of genetic polymorphisms, weight, or age on route efficiency.

Given the very small sample (*n* = 20), this analysis is likely underpowered to detect small-to-moderate genetic effects; accordingly, we interpret the null result as inconclusive rather than evidence of absence and recommend caution in its interpretation.

## 4 Discussion

This study aimed to test for associations between polymorphisms of three genes, *DRD4, TPH2,* and *LDHA*, and behavioural variations in boldness, stress response, and navigation in homing pigeons. *DRD4* and *TPH2* showed significant associations with both boldness and stress response and *LDHA* showed significant association with stress response, highlighting the likelihood of a genetic basis for variation in behavioural phenotypes. We found significant repeatability in emergence likelihoods from a shelter in 10 min, in line with several previous studies [13,63] showing that inter-individual variation in this behaviour is a reliable measure of boldness. By contrast, the continuous latency measure was not significantly repeatable, which might mainly reflect a modest sample size for the continuous metric, right-censoring at 600 s, as we have ruled out potential learning or habituation by including trial as a confounding factor in our models [64]. Repeatability in a trait sets the upper limit of heritability under certain conditions [65], therefore it is promising to look at the heritable component that may underlie the repeatability in behaviour. The findings will be valuable for future studies exploring the fitness trade-offs associated with different behavioural phenotypes. Such studies can also address a key limitation of comparative analyses, namely that developmental history may strongly influence behavioural phenotypes and thus confound comparisons [66].

Our study has shown that boldness is associated with genetic polymorphisms at both *DRD4* and *TPH2*. In *DRD4*, pigeons with genotype T/T emerged from shelter faster and were bolder in novel environments, consistent with its dopaminergic role in novelty seeking. Therefore, the allele frequency distribution of C and T may have resulted in the spectrum of shyness and boldness observed in our population. Similarly, in *TPH2*, the heterozygous genotype T/A was most associated with increased boldness in homing pigeons. In contrast, there was no discernible effect of genetic polymorphisms at *LDHA* on the results of our behavioural assays. These findings complement the increasing evidence that *DRD4* and *TPH2* play a role in the genetic architecture of exploration behaviour. Behavioural and personality traits are frequently associated with individual fitness [[67], [68], [69]], raising the question of why persistent interindividual variation in behavioural phenotypes and their associated genotypes is maintained in natural populations. One proposed explanation is that evolutionary trade-offs and frequency-dependent selection contribute to the stability of this variation [69]. For instance, pleiotropic effects of genes may give rise to behavioural syndromes, whereby multiple behaviours are subject to correlational selection due to shared genetic architecture. Consequently, functionally independent traits, such as novelty-seeking and stress responsiveness may be co-selected under common ecological pressures, such as predation risk [70]. Our results, together with other studies on the relationships between *DRD4* and behaviour in other avian species [71], indicate that such genes could generate suites of correlated behaviours with potential evolutionary consequences. Interestingly, these behavioural syndromes may represent alternative adaptive strategies, whose relative fitness benefits depend on the environment and its variability across spatial and temporal scales [70,72,73]. Moreover, the presence of multiple personality types within social groups may modulate group-level dynamics and influence collective outcomes [13]. Investigating the genetic underpinnings of behaviour can therefore help understand the evolutionary mechanisms driving and maintaining behavioural heterogeneity.

Previous studies on a repertoire of behavioural assays (including exploratory tendency) have shown that personality in captivity, as in our study, is likely to reflect that in the wild [74]. Nonetheless, we would still recommend the development of genotype-phenotype studies in natural populations to understand the genetic driving forces of behavioural traits that are shaped by natural selection within wild populations. At the same time, we would also recommend follow-up research to investigate the effect of different settings for emergence test on boldness assays [75].

In our study, we also found positive correlations between genetic polymorphisms in all three of our candidate genes and response to stress. In *DRD4*, genotype C/C showed a slower increase in eye temperature compared to the other two genotypes (C/T and T/T), implying a slower recovery from stress. The C/C genotype was also found to correlate with a shy phenotype, thus suggesting boldness and stress response have a common genetic control. Conversely, in *TPH2*, genotype T/A was also associated with both boldness and slow stress recovery, which might indicate a heterozygous trade-off between exploration and stress resilience. While the effect of *DRD4* on resilience to stresses has been studied in humans [76], its study in birds is still confined to novelty-seeking behaviour. Similarly, many studies have suggested a role for *TPH2* in modulating personality traits of negative emotionality in humans and model organisms [46,[77], [78], [79]], and our study suggests a potentially similar mechanism in birds, expanding the suite of candidate genes for behavioural screening. The case for *LDHA* is not as straightforward: while there is a significant effect on mirror*genotype interaction, such that ML (medium-long), MM (medium-medium), SL (short-long), and SS (short-short) all have a higher eye temperature when a mirror is present, while LL (long-long) and SM (short-medium) have the opposite. There is no clear trend where the shorter the repeats, the quicker the recovery from stress. As a critical glycolytic enzyme, *LDHA* level elevates to enhance glycolysis and cellular hypertrophic growth in response to stress, as found in humans [80] and mice [81]. This genotype-dependent perception of social cues in stress modulation is new to report. Our interpretation remains provisional and warrants further investigation.

Using eye region surface temperature as an indicator of stress is a relatively recent advancement, applied in research on domesticated chickens [82] and blue tits [83,84]. Studies by Herborn et al. [76] and Jerem et al. [77] have shown that acute stress leads to a decrease in peripheral surface temperature, including the eye region, likely due to vasoconstriction, followed by a gradual recovery as the stress subsides. In our study, we observed a gradual increase in eye temperature over the 10-minute isolation period, which we interpret as recovery from initial acute stress associated with transfer and subsequent habituation to the novel environment. Accordingly, birds that exhibited faster increases in eye temperature may have experienced lower or shorter-lasting stress, consistent with the genotype-related differences observed. While this pattern is consistent with prior thermography work, we did not obtain a post-habituation resting baseline immediately before isolation, and thus this inference should be regarded as cautious rather than definitive. We use the term “stress recovery” to describe this re-warming trajectory pragmatically, and we recommend future work include a clear post-habituation baseline and concurrent physiological measures such as heart rate variability to strengthen validation of eye-temperature recovery as a proxy for stress resilience.

The effect of the mirror, intended to provide visual social cues and hence reduce neophobia in a novel environment, on stress recovery was small but not negligible. However, we still have limited understanding of pigeons’ perception towards mirror images. Pigeons do not show spontaneous mirror self-recognition, but can be conditioned for food reward to pass the mirror test [85]. A previous study shows that they treat mirror images as uncanny competitors, but in the context of food foraging [60], which is deemed a highly stressful situation for birds in general [86]. However, a conspecific in a neophobic context without food competition can in turn facilitate stress remediation [27], as mirrors have been shown to mitigate isolation-related stress in other birds [61] and have similar benefits comparable to conspecific playback [62]. We have also shown how different genotypes might have differential benefits from mirror-induced stress mitigation, which is likely to again modulate group-level dynamics and fitness in a social bird. We suggest caution in the interpretation of pigeons’ perception towards mirrors and suggest future studies using live conspecifics or video playback to quantify the strength of social buffering across modalities.

Although we found no significant effect of genetic polymorphisms on large-scale homing performance, the limited sample size used in the analysis, constrained by having to use a previously gathered, small dataset – due to our subjects being confined to the lofts as part of Avian Influenza preventative measures for the duration of this study – means that we cannot at present be confident in our conclusion. In previous studies, *DRD4* was identified as potentially associated with superior performance in short-distance races [87], and *LDHA* was found to contribute to flight energetics by prolonging muscle endurance [50,88]. Our limitations also address the necessity of large sample sizes in genetic association studies to reduce the sample error.

Many previous studies, including ours, utilize the genetic information embodied by the SNP as a dimension of genetic polymorphism. SNP is regarded as an effective marker in studying genetic polymorphism in various animal populations in both candidate gene approaches and genome-wide association studies [[89], [90], [91], [92], [93]]. We confirm the utility of genetic polymorphisms as a powerful tool to decipher differential behavioural and cognitive phenotypes. The approach is also noted as an important part of functional genomics and linkage analysis studies. With the advancement of sequencing technology, we expect that SNP discovery will become increasingly efficient and accompany more genetic studies in the near future, shedding more light onto avian behaviour, cognition, and evolution.

## 5 Conclusion

We have presented evidence demonstrating that genetic polymorphism at *DRD4* and *TPH2* is associated with variations in boldness and stress response in birds, using homing pigeons as our model. Such variation is independent of physical attributes including age, weight, and sex, is found to be repeatable and consistent, and may be considered as a potential genetic marker for the study of the genetics of pigeons’ personality. We must note that the associated coding SNPs in *DRD4* and *TPH2* are synonymous and the short-tandem repeat in *LDHA* is intronic, thus any effects are likely mediated by expression-level mechanisms such as codon-usage-dependent translation dynamics, mRNA secondary structure, or splicing regulation, rather than by protein sequence changes [94,95], which warrants further mechanistic and functional studies. Also, non-candidate, unlinked markers may be used to stratify the association between candidate markers and phenotype. Our study presents a promising direction for elucidating the genetic basis of various dimensions of avian behaviour and personality, which may be acted on by natural selection and hence drive lineage divergence in avian evolution. As an auxiliary finding, our study also shows the antagonistic effect of social detachment and social buffering on stress response and recovery in birds. This opens up exciting possibilities for follow-up research on the effect of social context on stress physiology and behaviour. In sum, although our results with respect to navigational performance remain inconclusive, our work presents a multi-faceted study that addresses some important but little-explored questions in seeking the genetic basis of avian behavioural variation. We are confident that more detailed investigations will follow in the near future. Future work combining gene expression assays, pharmacological manipulations, or experimental crosses could test causal pathways suggested by these associations.

## Data availability statement

All data and scripts used in this study have been deposited onto Dryad (https://doi.org/10.5061/dryad.v6wwpzh8d).

## Ethics statement

All methods employed in this study were approved by the Ethical Review Committee of the Department of Zoology, University of Oxford (reference number APA/1/5/ZOO/NASPA/Biro).

## CRediT authorship contribution statement

**Sherif I. Ramadan:** Writing – review & editing, Methodology, Investigation, Formal analysis, Data curation, Conceptualization, Visualization. **Tin Hang Hung:** Writing – original draft, Methodology, Investigation, Formal analysis, Data curation, Conceptualization, Funding acquisition, Visualization. **Mathilde Delacoux:** Writing – review & editing, Visualization, Validation, Methodology, Investigation, Formal analysis, Conceptualization. **Fumihiro Kano:** Writing – review & editing, Validation, Supervision, Methodology. **Miho Inoue-Murayama:** Writing – review & editing, Supervision, Project administration, Methodology, Funding acquisition. **Dora Biro:** Writing – review & editing, Supervision, Project administration, Methodology, Funding acquisition.

## Declaration of competing interest

The authors declare no conflict of interest. The funders had no role in the design of the study; in the collection, analyses, or interpretation of data; in the writing of the manuscript; or in the decision to publish the results.

## Appendix Supplementary materials

Image, application 1
