# Supplementary material for: Associations of genetic polymorphisms with boldness, stress response, and route efficiency in homing pigeons (Columba livia)
Source: Physiol Behav. 2026 Mar 1;305:115211. doi: 10.1016/j.physbeh.2025.115211 (PMC13328059; doi:10.1016/j.physbeh.2025.115211)
Supplement: Supplementary file 1 [file mmc1.docx]

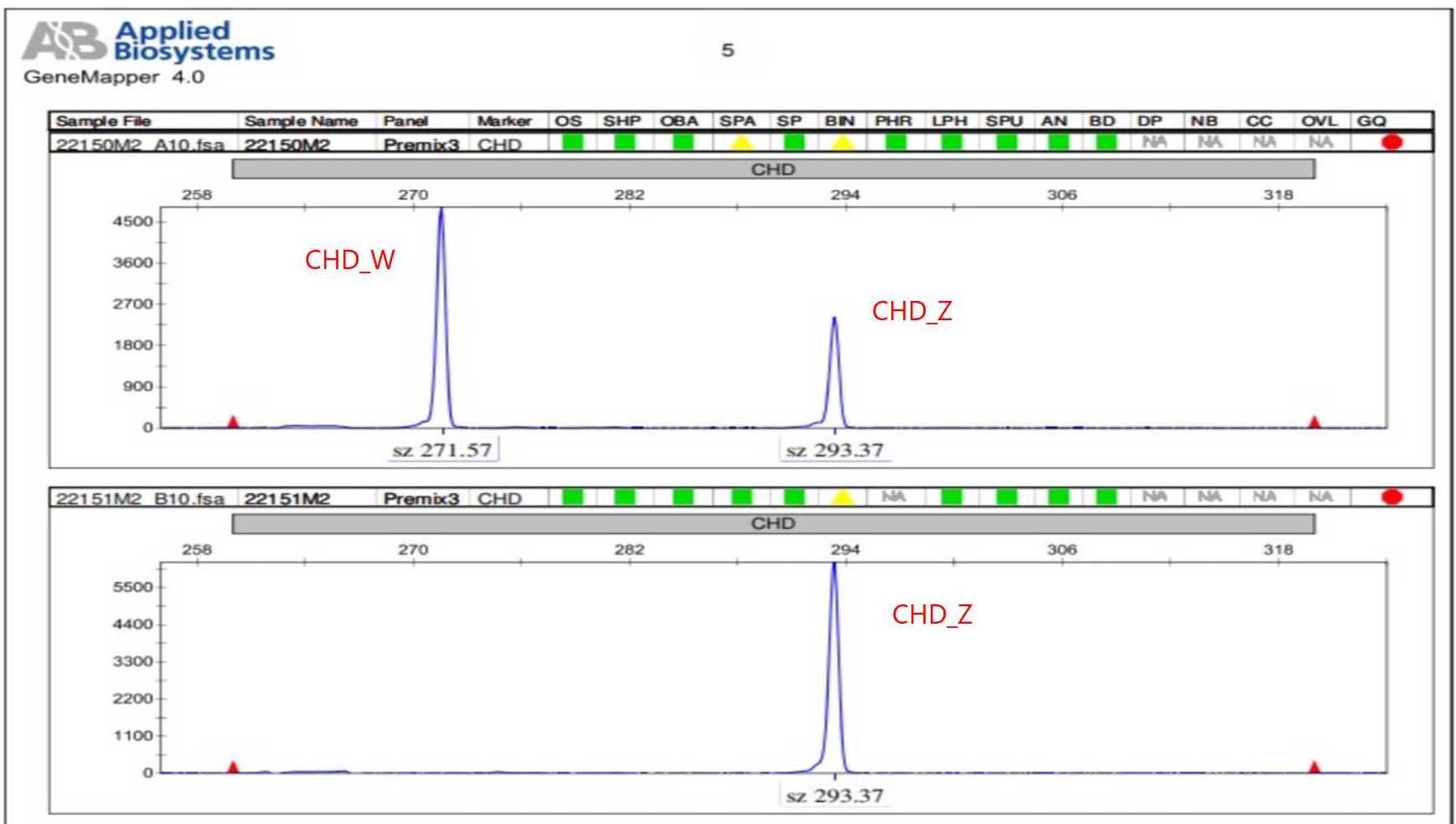


**Supplementary Figure 1**. Visualisation of genotyping results for CHD, showing heterozygous W/Z alleles and homozygous Z alleles.


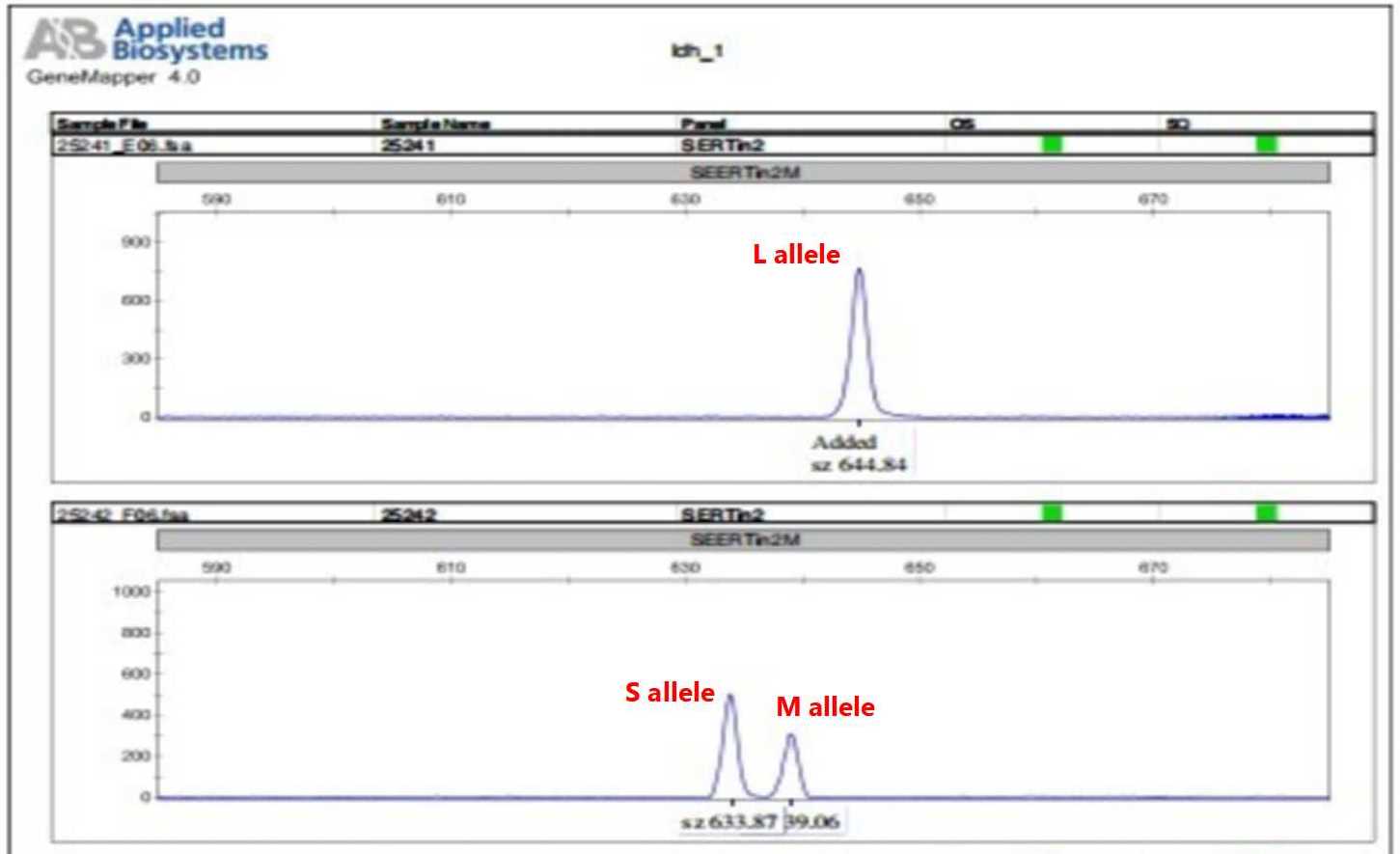


**Supplementary Figure 2**. Visualisation of genotyping results for LDHA, showing S (short), M (middle), and L (long) alleles.

**Supplementary Table 1**. Summary descriptive statistics of different genotypes of (a) *DRD4*, (b) *TPH2*, and (c) *LDHA* in boldness, stress, and navigation tests.

(a)

|  | **DRD4** | | |
| --- | --- | --- | --- |
|  | **C/C** | **C/T** | **T/T** |
| **Age (mean, years)** | 4.250000 | 5.030769 | 4.821429 |
| **Age (SD)** | 2.720294 | 3.749872 | 3.63106 |
| **Weight (mean, grams)** | 449.6429 | 482.5283 | 487.9178 |
| **Weight (SD)** | 48.76097 | 40.59301 | 46.61418 |
| **Boldness trial 1 (mean, seconds)** | 490.2308 | 432.2642 | 365.2239 |
| **Boldness trial 1 (SD)** | 202.9972 | 228.0756 | 250.6500 |
| **Boldness trial 2 (mean, seconds)** | 489.3571 | 418.4717 | 381.2647 |
| **Boldness trial 2 (SD)** | 202.9819 | 236.6224 | 254.8058 |
| **Boldness overall (mean, seconds)** | 493.7143 | 425.3679 | 371.2794 |
| **Boldness overall (SD)** | 118.1207 | 185.5708 | 209.3726 |
| **Stress test, no mirror (mean, °C)** | -0.006670 | 0.375096 | 0.427348 |
| **Stress test, no mirror (SD)** | 0.484061 | 0.593910 | 0.481647 |
| **Stress test, with mirror (mean, °C)** | 0.035000 | 0.530816 | 0.516000 |
| **Stress test, with mirror (SD)** | 0.503877 | 0.486728 | 0.548145 |
| **Navigation speed (mean, m/s)** | 3.540136 | 3.570222 | 3.563889 |
| **Navigation speed (SD)** | 0.138311 | 0.150432 | 0.181952 |
| **Navigation IRE (mean)** | 0.288429 | 0.363808 | 0.411911 |
| **Navigation IRE (SD)** | 0.095654 | 0.247679 | 0.172080 |

(b)

|  | **TPH2** | | |
| --- | --- | --- | --- |
|  | **A/A** | **T/A** | **T/T** |
| **Age (mean, years)** | 4.526316 | 4.972222 | 4.909091 |
| **Age (SD)** | 3.761374 | 3.455847 | 3.693201 |
| **Weight (mean, grams)** | 493.8148 | 488.8000 | 466.2917 |
| **Weight (SD)** | 39.41011 | 41.43527 | 50.82090 |
| **Boldness trial 1 (mean, seconds)** | 432.9630 | 403.3279 | 388.0000 |
| **Boldness trial 1 (SD)** | 256.6522 | 236.6843 | 237.2994 |
| **Boldness trial 2 (mean, seconds)** | 494.2593 | 363.6721 | 413.3404 |
| **Boldness trial 2 (SD)** | 205.7292 | 259.2474 | 232.6194 |
| **Boldness overall (mean, seconds)** | 463.6111 | 383.5000 | 399.8404 |
| **Boldness overall (SD)** | 181.4579 | 196.1657 | 199.5366 |
| **Stress test, no mirror (mean, °C)** | 0.479038 | 0.267167 | 0.435114 |
| **Stress test, no mirror (SD)** | 0.455333 | 0.530219 | 0.584019 |
| **Stress test, with mirror (mean, °C)** | 0.632500 | 0.365452 | 0.528712 |
| **Stress test, with mirror (SD)** | 0.531295 | 0.481561 | 0.591679 |
| **Navigation speed (mean, m/s)** | 3.531384 | 3.518208 | 3.603931 |
| **Navigation speed (SD)** | 0.184596 | 0.220418 | 0.107560 |
| **Navigation IRE (mean)** | 0.475624 | 0.310913 | 0.362564 |
| **Navigation IRE (SD)** | 0.152787 | 0.157765 | 0.235593 |

(c)

|  | **LDHA** | | | | | |
| --- | --- | --- | --- | --- | --- | --- |
|  | **LL** | **ML** | **MM** | **SL** | **SM** | **SS** |
| **Age (mean, years)** | 5.600000 | 5.194444 | 4.512195 | 4.000000 | 5.972222 | 3.521739 |
| **Age (SD)** | 2.221111 | 3.639619 | 3.186863 | 2.285218 | 4.919269 | 2.556090 |
| **Weight (mean, grams)** | 472.7778 | 468.1290 | 482.6944 | 486.5333 | 482.3548 | 505.1111 |
| **Weight (SD)** | 50.52914 | 42.47567 | 44.31692 | 54.40046 | 44.70835 | 41.23803 |
| **Boldness trial 1 (mean, seconds)** | 378.3750 | 403.9667 | 389.7059 | 445.0000 | 380.2000 | 454.1765 |
| **Boldness trial 1 (SD)** | 244.3685 | 230.4753 | 247.8105 | 221.2764 | 254.5440 | 248.7029 |
| **Boldness trial 2 (mean, seconds)** | 384.1250 | 450.2581 | 399.2353 | 334.2000 | 393.5667 | 443.0000 |
| **Boldness trial 2 (SD)** | 236.6634 | 229.3466 | 245.8113 | 244.5334 | 262.3392 | 246.9714 |
| **Boldness overall (mean, seconds)** | 381.2500 | 430.2742 | 394.4706 | 378.0333 | 386.8833 | 448.5882 |
| **Boldness overall (SD)** | 177.7687 | 173.6658 | 198.3098 | 191.3078 | 200.5137 | 241.7864 |
| **Stress test, no mirror (mean, °C)** | 0.702857 | 0.339286 | 0.253971 | 0.386667 | 0.347167 | 0.522500 |
| **Stress test, no mirror (SD)** | 0.898772 | 0.495363 | 0.486308 | 0.402646 | 0.633323 | 0.427153 |
| **Stress test, with mirror (mean, °C)** | 0.321875 | 0.568966 | 0.560167 | 0.450333 | 0.257321 | 0.597941 |
| **Stress test, with mirror (SD)** | 0.471350 | 0.561855 | 0.517661 | 0.379306 | 0.484473 | 0.691712 |
| **Navigation speed (mean, m/s)** | 3.690508 | 3.515127 | 3.469218 | 3.727559 | 3.479938 | 3.498614 |
| **Navigation speed (SD)** | 0.067297 | 0.134348 | 0.186142 | 0.145498 | 0.223444 | NA |
| **Navigation IRE (mean)** | 0.475585 | 0.365126 | 0.293081 | 0.389866 | 0.460818 | 0.168042 |
| **Navigation IRE (SD)** | 0.412111 | 0.151477 | 0.313215 | 0.097527 | 0.148142 | NA |
